# Supercharged Natural Killer (sNK) Cells Inhibit Melanoma Tumor Progression and Restore Endogenous NK Cell Function in Humanized BLT Mice

**DOI:** 10.3390/cancers17152430

**Published:** 2025-07-23

**Authors:** Kawaljit Kaur, Paytsar Topchyan, Anahid Jewett

**Affiliations:** 1 Division of Oral Biology and Oral Medicine, The Jane and Jerry Weintraub Center for Reconstructive Biotechnology, UCLA School of Dentistry and Medicine, 10833 Le Conte Ave, Los Angeles, CA 90095, USA; paytsar.topchyan@ucla.edu (P.T.); ajewett@mednet.ucla.edu (A.J.); 2 The Jonsson Comprehensive Cancer Center, University of California, Los Angeles, CA 90024, USA

**Keywords:** NK cells, supercharged NK cells, cytotoxicity, melanoma, IFN-γ, humanized BLT mice, cancer stem cells (CSCs), immunotherapy

## Abstract

Background: We have previously shown the remarkable impact of a single infusion of supercharged NK cells (sNK) in preventing and eliminating oral, pancreatic, and uterine cancers implanted in humanized BLT (hu-BLT) mice. Objective: In this report, we extended the studies to melanoma tumors to observe whether there were differences in response to sNK cells. Methods: We investigated the safety and tissue biodistribution profile of sNK cells in hu-BLT mice. This included the effect of sNK cell therapy on the peripheral blood-derived PBMCs, bone marrow, and spleen of hu-BLT mice. Results: Our investigation showed promising outcomes, as sNK cell infusions effectively inhibited melanoma tumor growth in hu-BLT mice. These potent cells not only traversed through the peripheral blood, spleen, and bone marrow but also infiltrated the tumor site, triggering in vivo differentiation of melanoma tumors. Moreover, the infusion of sNK cells increased the percentages of NK cells in the peripheral blood of hu-BLT mice, restoring cytotoxicity and IFN-γ secretion within the peripheral blood, spleen, and bone marrow of melanoma-bearing mice. Conclusions: This therapeutic approach not only reversed tumor progression but also revitalized the functionality of endogenous NK cells, potentially reversing the immunosuppressive effects induced by tumor cells in cancer patients.

## Simple Summary

In this study, we investigated the utilization of supercharged NK cells to treat melanoma in humanized mice. Humanized mice were developed by implanting human fetal tissues into immunocompromised mice; the immune cells of humanized mice closely resemble those of humans, with minimal differences. The investigation examined tumor growth, biodistribution of infused supercharged NK cells in mice tissues and tumors, and the immune cell functionality in humanized mice post supercharged NK cell infusions. The findings revealed the potential of supercharged NK cell-based therapies in treating melanoma and enhancing the immune system to establish long-term immunity in cancer patients. The study demonstrated that administering supercharged NK cells resulted in heightened NK cell proportions, increased cytokine release, and enhanced cytotoxic capabilities of immune cells in peripheral blood-derived mononuclear cells, the spleen, and bone marrow of mice with tumors in comparison to the untreated group. Moreover, tumor-bearing mice treated with supercharged NK cells exhibited increased IFN-γ secretion and enhanced cytotoxicity of immune cells in various tissues, surpassing the levels observed in the untreated group. This research sheds light on the promising prospects of supercharged NK cell-based interventions for melanoma treatment, showcasing advancements in immune response amplification and strategies to combat tumors.

## 1. Introduction

Melanoma, the fifth most common cancer in the United States, is still increasing both nationally and globally, representing 3% of skin cancers, but is responsible for 65% of skin cancer-related deaths [1,2,3,4]. The primary treatment approach for melanoma is surgery, but chemotherapy shows prognostic benefits for melanoma patients [2,5]. Recent advancements in immunotherapies, such as immune checkpoint inhibitors and IL-2-activated T cells, have significantly improved the overall survival rates of melanoma patients [6,7,8,9]. Cancer stem cells (CSCs) play a pivotal role in the aggressive and chemoresistant nature of melanoma [10,11,12]. Melanoma-related malignant lesions originate from rapid multiplication of skin cancer due to mutations/genetic defects caused by unrepaired DNA damage of skin resulting from ultraviolet radiation from sunlight or tanning beds [4,13,14].

Natural killer (NK) cells are pivotal in inhibiting cancer through various effector functions. These include direct cytotoxicity against tumors, antibody-dependent cellular cytotoxicity (ADCC), and the indirect modulation of other immune effectors by secreting inflammatory cytokines and chemokines [15,16,17]. NK cells have been found to recognize and target CSCs that express lower levels of MHC-class I, CD54, and PD-L1 (B7H1) receptors and higher levels of CD44 [18,19]. Our research has shown a strong association between tumor differentiation stages and their vulnerability to primary NK cell-mediated cytotoxicity [20].

The healthy skin immune cell population includes NK cells that exhibit cytotoxic function against stressed or infected melanoma-like cells [21,22]. Studies have shown that the presence of skin fibroblasts can differentiate purified CD56^bright^CD16^dim^ NK cells into CD56^dim^ NK cells with characteristics and phenotype similar to peripheral blood CD56^dim^ NK cells [23]. Studies have linked decreased NK cell activity to a negative prognosis in cancer patients [24,25,26,27,28,29,30,31,32,33,34,35,36,37,38,39,40,41,42,43,44,45,46,47,48,49]. The effectiveness of NK cells in treating solid tumors is widely acknowledged [50,51,52,53,54,55]. Melanoma cell lines have been found to exhibit increased levels of NKG2D ligands MICA/B, which play a crucial role in activating NK cells during interactions with melanoma cells [56,57]. Ligands for NK cell receptors Nkp44, Nkp46, and Nkp30 have also been found to be expressed in melanoma cells [58,59]. Ongoing research underscores the promising potential of NK cell-based therapies for melanoma treatment

This study was conducted to evaluate the effectiveness of supercharged NK (sNK) cells in treating melanoma in humanized BLT mice (hu-BLT). sNK cells were adoptively transferred to hu-BLT mice with melanoma. The process of sNK generation involved co-culturing NK cells with osteoclasts (OCs) and seven strains of sonicated probiotic bacteria. The interaction of surface markers of NK cell receptor ligands and secreted factors of OCs played a crucial role in expanding and activating NK cells [60]. Treatment with the selected probiotic bacterial strains enhanced NK cell activation and cytokine secretion [61,62]. Combining OCs and probiotics led to the activation of signals that promoted NK cell expansion and functional activation. Our previous studies have revealed that sNK cells exhibit increased cell expansion, cytotoxicity, and cytokine secretion. Moreover, sNK cells demonstrate an extended lifespan and the ability to induce tumor differentiation both in vivo and in vitro [61]. sNK cells have shown enhanced survival in the tumor microenvironment (TME) due to elevated levels of anti-apoptotic proteins like BCL2 and reduced levels of pro-apoptotic proteins, enabling them to resist tumor-induced cell death [63]. Increased expression levels of cytotoxic-associated granules and Trail contribute to increased cytotoxic function in sNK cells [63]. Furthermore, sNK cells display enhanced regulatory function; the majority of an sNK cell population was found during the active cycling phase, and sNK cells express increased proliferation-associated genes and proteins, along with higher levels of memory-associated genes [63]. Most importantly, sNK cells show elevated expressions of activating receptors such as CD16, CD56, Nkp30, Nkp44, Nkp46, NKG2D, CD94, CD54, KIR2, KIR3, and 4-1BBL while demonstrating decreased expression of inhibitory receptors like NKG2A, PD-1, and TIGIT [61,63]. Detailed data on mitochondrial profiling; transcriptomic, genomic, and memory phenotypes; and markers associated with persistence were demonstrated in our previous publication [63].

Our previous research findings reveal the superior anti-cancer activity of sNK cells over various other NK cell treatments. Comparisons with IL-2, IL-2 + anti-CD16 mAbs, IL-2 + anti-CD16 mAbs + sAJ2, IL-12, IL-15, IL-18, OSCSCs, and K562-expanded NK cells demonstrate the remarkable anti-cancer functions of sNK cells [61,64,65,66]. In addition, when compared to cord blood-derived NK cells, iPSC-derived NK cells, NK92 cells, and several other NK cells, sNK cells have exhibited much higher levels of cytotoxicity and cytokine secretion [67,68].

In this study, we found a significantly reduced tumor mass and increased survival in sNK cell-treated mice in comparison with untreated melanoma-bearing hu-BLT mice. Data showed a notable increase in the percentage of NK cells, elevated secretion of IFN-γ, and improved cytotoxic capabilities of immune cells in various body tissues of tumor-bearing mice following treatment with sNK cells. Specifically, peripheral blood-derived mononuclear cells (PBMCs), spleen, and bone marrow exhibited heightened immune responses compared to untreated tumor-bearing mice. Moreover, the infusion of sNK cells also led to enhanced IFN-γ secretion and cytotoxic functions in additional tissues of tumor-bearing mice, underscoring the positive impact of sNK cells as a treatment approach for melanoma.

## 2. Materials and Methods

### 2.1. Cell Lines, Reagents, and Antibodies

RPMI 1640 (Gibco, ThermoFisher, Waltham, CA, USA) supplemented with 10% Fetal Bovine Serum (FBS) (Gemini Bio-Products, West Sacramento, CA, USA) was used for the cultures of human NK cells and immune cells of hu-BLT mice. Recombinant IL-2 (rhIL-2) was obtained from PeproTech (Cranbury, NJ, USA). Flow cytometry and other antibodies used in the study were obtained from BioLegend (San Diego, CA, USA). Human melanoma A375 cells were donated by Dr. Toru Hiraga (Department of Histology and Cell Biology, Matsumoto Dental University, Japan) [69] and were cultured in DMEM (Life Technologies, Carlsbad, CA, USA) supplemented with 10% FBS and 2% antibiotic–antimycotic (Gemini Bio-Products). Oral squamous carcinoma stem cells (OSCSCs) were isolated from patients with tongue tumors at UCLA. OSCSCs were cultured in RPMI 1640 medium supplemented with 10% FBS, 2% Antibiotic/Antimycotic Solution (Cytiva, Marlborough, MA, USA), 1.4% Sodium Pyruvate (Gibco, Waltham, CA, USA), 1.4% MEM Non-Essential Amino Acids (Gibco, CA, USA), and 0.15% sodium bicarbonate. Human NK cells, T cells, and monocyte purification kits were obtained from Stem Cell Technologies (Vancouver, BC, Canada). The PKH26 staining kit was purchased from Sigma-Aldrich (St, Louis, MO, USA). Human ELISA kits for IFN-γ were purchased from Biolegend (San Diego, CA, USA). A multiple-panel analysis instrument was purchased from MAGPIX, Millipore (Billerica, MA, USA). Chromium-51 was purchased from PerkinElmer (Shelton, CT, USA).

### 2.2. Purification of Human NK Cells and Monocytes

Written informed consents approved by the UCLA Institutional Review Board (IRB) were obtained, and the UCLA-IRB approved all procedures. NK cells and monocytes were negatively selected from PBMCs using an EasySep^®^ Human NK cell enrichment kit and monocyte isolation kit, respectively, purchased from Stem Cell Technologies (Vancouver, BC, Canada). Isolated NK cells and monocytes were stained with anti-CD16 and anti-CD14 antibodies, respectively, to measure the cell purity using flow cytometric analysis.

### 2.3. Probiotic Bacteria AJ2 Sonication

Gram-positive probiotic bacteria: AJ2 bacteria (*Streptococcus thermophilus*, *Bifidobacterium longum*, *Bifidobacterium breve*, *Bifidobacterium infantis*, *Lactobacillus acidophilus*, *Lactobacillus plantarum*, and *Lactobacillus paracasei*) were weighed and resuspended in RPMI 1640 containing 10% FBS at a concentration of 10 mg/1mL. The bacteria were thoroughly vortexed, then sonicated on ice for 15 s, set at a 60% amplitude. Sonicated samples were then incubated for 30 s on ice. After every five pulses, a sample was taken for observation under a microscope until at least 80 percent of the bacterial cell walls were lysed. It was determined that approximately 20 rounds of sonication/incubation on ice were needed to achieve complete sonication. Finally, the sonicated sAJ2 samples were aliquoted and stored in a −80 °C freezer.

### 2.4. Generation of Osteoclasts and Expansion of Human NK Cells

Purified monocytes from human peripheral blood were cultured in alpha-MEM medium containing M-CSF (25 ng/mL) and RANKL (25 ng/mL) for 21 days unless otherwise specified. The medium was refreshed every 3 days with fresh alpha-MEM containing M-CSF and RANKL. Human purified NK cells were activated with rh-IL-2 (1000 U/mL) and anti-CD16mAb (3 μg/mL) for 18–20 h before they were co-cultured with osteoclasts and sonicated AJ2 for NK cell expansion (NK:OCs:sAJ2; 2:1:4). The medium was refreshed every 3 days with RMPI containing rh-IL-2 (1500 U/mL).

### 2.5. Analysis of Melanoma Growth in Humanized BLT Mice

Animal research was performed under the written approval of the UCLA Animal Research Committee (ARC). Humanized BLT (hu-BLT; human bone marrow/liver/thymus) mice were generated as previously described [70,71]. For in vivo melanoma growth, A375 human melanoma cells (1 × 10^6^) were mixed 1:1 with HC Matrigel (Corning, Corning, NY, USA) and injected subcutaneously into the right flank of hu-BLT mice as described previously [71,72]. Tumor growth was measured biweekly with a caliper. The sNK cell therapy group received four weekly tail-vein injections of 2 × 10^6^ sNK cells. Mice were euthanized when signs of morbidity were evident. Melanoma tumor, bone marrow, spleen, and peripheral blood samples were harvested from mice at the end of the experiment or when the tumor size reached 2 cm in diameter.

### 2.6. Cell Dissociation and Cell Culture of Tissues from Hu-BLT Mice

The melanoma tumor samples harvested from hu-BLT mice were immediately cut into 1 mm^3^ pieces and placed into a digestion buffer containing 1 mg/mL collagenase IV, 10 U/mL DNAse I, and 1% bovine serum albumin (BSA) in DMEM media and incubated for 20 min at 37 °C in an oven on a 150 rpm shaker. After digestion, the sample was filtered through a 40 mm cell strainer and centrifuged at 1500 rpm for 10 min at 4 °C. The pellet was resuspended in DMEM media, and cells were counted. To obtain single-cell suspensions from BM, femurs were cut from both ends and flushed from one end to the other using RPMI media, and BM cells were filtered through a 40 µm cell strainer. To obtain single-cell suspensions from the spleen, the spleen was smashed until no big piece was left, and the sample was filtered through a 40 µm cell strainer and centrifuged at 1500 rpm for 5 min at 4 °C. The pellet was re-suspended in ACK buffer for 2–5 min to remove the red blood cells, followed by re-suspension in RMPI media and centrifuged at 1500 rpm for 5 min at 4 °C. Peripheral blood mononuclear cells (PBMCs) were isolated using Ficoll–Hypaque centrifugation of heparinized blood specimens. The buffy coat containing PBMCs was harvested, washed, and resuspended in RPMI 1640 medium.

### 2.7. Purification of NK Cells and CD3+ T Cells from Hu-BLT Mice

NK cells and CD3+ T cells from hu-BLT mouse splenocytes were isolated using a human CD56+ selection kit and human CD3+ T selection kit, respectively (Stem Cells Technologies, Vancouver, CA, Canada).

### 2.8. Enzyme-Linked Immunosorbent Assays (ELISAs) and Multiplex Cytokine Assay

The assay was conducted as described in the manufacturer’s protocol. The plates were read in a microplate reader at 450 nm to obtain absorbance values (Biolegend, ELISA manual). To analyze and obtain the cytokine and chemokine concentration, a standard curve was generated by either two- or three-fold dilution of recombinant cytokines provided by the manufacturer.

A multiplex assay was conducted as described in the manufacturer’s protocol for each specified kit. Analysis was performed using a Luminex multiplex instrument (MAGPIX, Millipore, Billerica, MA, USA), and data was analyzed using the proprietary xPONENT 4.2 software (Millipore, Billerica, MA, USA).

### 2.9. Surface Staining Assays

Staining of the cells was conducted by labeling them with specific antibodies following a standard protocol as described previously [73]. Briefly, the cells were washed twice with ice-cold PBS/1% BSA. Predetermined optimal concentrations of specific human flow cytometric antibodies were added to 1 × 10^4^ cells in 50 µL of ice-cold PBS/1%BSA, and cells were incubated on ice for 30 min. Thereafter, cells were washed in cold PBS/1%BSA and brought to 500 µL with PBS/1%BSA. Flow cytometric analysis was performed using a Beckman Coulter Epics XL cytometer (Brea, CA, USA), and results were analyzed using FlowJo vX10 software (Ashland, OR, USA).

### 2.10. ^51^Cr Release Cytotoxicity Assay

The ^51^Cr release assay was performed as described previously. We used OSCSCs as target cells to evaluate NK cell-mediated cytotoxicity due to OSCSCs’ high susceptibility to NK cell-mediated cytotoxicity. Briefly, different ratios of effector cells were incubated with ^51^Cr-labeled target cells. After a 4 h incubation period, the supernatants were harvested from each sample and counted for released radioactivity using a gamma counter. The percentage specific cytotoxicity was calculated using the following formula:

% Cytotoxicity = Experimental cpm—spontaneous cpm                                  Total cpm—spontaneous cpm

Lytic unit 30/10^6^ was calculated by using the inverse of the number of effector cells needed to lyse 30% of the target tumor cells X100.

### 2.11. Statistical Analysis

For statistical analysis, an unpaired, two-tailed Student’s t-test was conducted, and Prism-10 software facilitated one-way ANOVA for group comparisons. Cultures were performed using duplicates or triplicates of samples, with the average used for each mouse. The notation of (n) indicates the number of mice utilized in each experimental condition. Statistical significance levels are denoted by **** (*p*-value < 0.0001), *** (*p*-value 0.0001–0.001), ** (*p*-value 0.001–0.01), and * (*p*-value 0.01–0.05).

## 3. Results

### 3.1. Safety and Biodistribution Profile of Supercharged NK Cells Infused into Healthy Hu-BLT Mice

**Figure 1:**
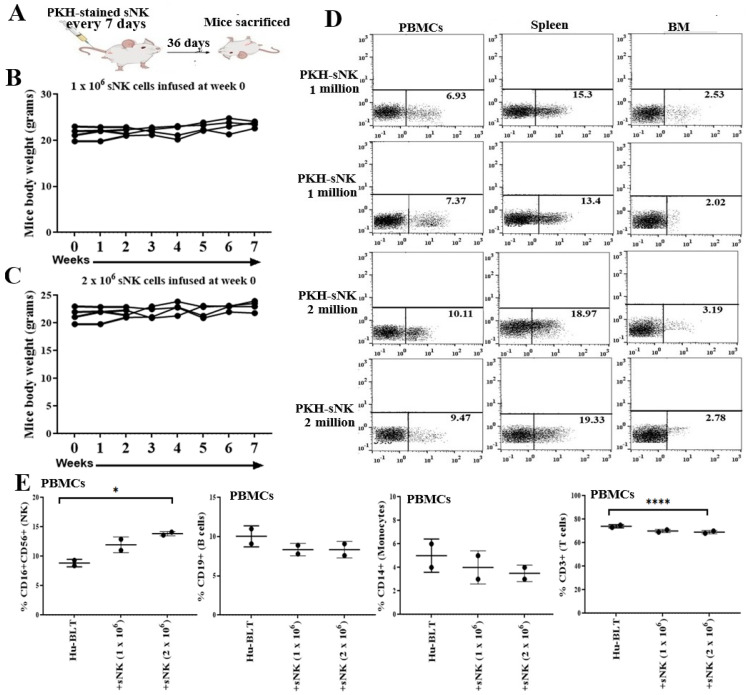
Biodistribution and safety of sNK cells in disease-free hu-BLT mice. Hu-BLT mice were administered PKH sNK cells via the tail vein (1 × 10^6^ and 2 × 10^6^ sNK cells in a total of 4 infusions), and mice were sacrificed 36 days after the first sNK cell infusion (**A**). The body weight of mice was recorded every week for 7 weeks (*n* = 4) (**B**,**C**). On day 36, mice were sacrificed; peripheral blood-derived PBMCs, splenocytes, and bone marrow cells were analyzed for PKH sNK cells (*n* = 2) (**D**); and PBMCs were analyzed for percentages of CD16+CD56+ NK cells, CD19+ B cells, CD14+ monocytes, and CD3+ T cells using flow cytometry (*n* = 2) (**E**). **** (*p*-value < 0.0001), and * (*p*-value 0.01–0.05).

We administered two different dose levels of sNK cells into healthy humanized BLT (hu-BLT) mice, and the mice were monitored for up to seven weeks (Figure 1A). Mice were monitored every 7 days, focusing on several health criteria, such as agility, body posture, coat condition, appetite, signs of organ swelling, skin lesions, and weight fluctuations. Throughout these studies, no indications of toxicity or adverse safety events, including pain, distress, or discomfort, were noted in mice that received sNK cells. Interestingly, the mice that received sNK cells exhibited higher levels of energy and activity compared to the control group. Not much change in mouse body weight was seen at either a 1 × 10^6^ or 2 × 10^6^ sNK cell dose during the 7-week monitoring period (Figure 1B,C).

Infused sNK cells circulated in the peripheral blood, spleen, and bone marrow of hu-BLT mice. The highest sNK cell biodistribution was seen in the spleen, followed by PBMCs, and the lowest was seen in the BM (Figure 1D). The findings suggest that sNK cells have a prolonged presence in the circulatory system and can migrate to key immune compartments. This ability of sNK cells to persist long after administration is crucial for their potential therapeutic effectiveness. Overall, the persistence and circulation of sNK cells demonstrate their viability for long-term immune modulation in vivo and highlighting their ability to persist and localize in key immune organs, such as the spleen and bone marrow, following adoptive transfer.

Four sequential infusions of sNK cells every 7 days increased the percentages of CD16+CD56+ NK cells, and the increase was significant with the infusion of 2 × 10^6^ sNK cells (Figure 1E). Slight decreases were seen in the percentages of CD19+ B cells and CD14+ monocytes (Figure 1E), and there were significant decreases in the percentages of CD3+ T cells with the infusion of 2 × 10^6^ sNK cells (Figure 1E). Previously, we observed increased CD8+T-cell percentages in hu-BLT tissues post sNK cell administration, suggesting that the increase in T cells shown in Figure 1E most likely corresponds to an increase in CD8+ T cells. Thus, sNK cell treatment increased endogenous NK cells, one of the mechanisms by which further progression of the tumor can be controlled by patients’ endogenous immune cells.

### 3.2. Supercharged NK Cells Induced Effects on the Immune Cell Function of Disease-Free Hu-BLT Mice

**Figure 2:**
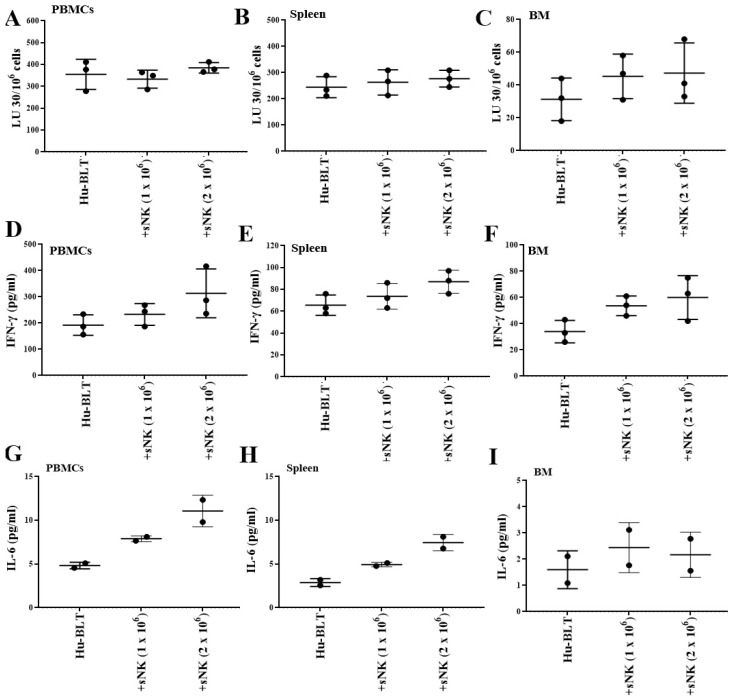
sNK cells induced effects on the immune cell function of disease-free hu-BLT mice. Hu-BLT mice were adoptively transferred with four sNK cell doses every 7 days: 1 × 10^6^ and 2 × 10^6^ sNK cells for 36 days (*n* = 2–3). Following sacrifice, peripheral blood, spleen, and BM samples were collected; single cell suspensions were prepared, treated with IL-2 (1000 U/mL), and cultured for 7 days; cytotoxicity assays were performed using hu-BLT mice cells as effectors in a standard 4 h ^51^Cr release assay against OSCSCs; and the LU 30/10^6^ cells were determined using an inverse number of cells required to lyse 30% of OSCSCs x100 (**A**–**C**). The supernatants from cultures were harvested, and the levels of IFN-γ (**D**–**F**) and IL-6 (**G**–**I**) were determined using specific ELISAs.

Four sequential infusions of sNK cells every 7 days induced no change or a slight increase in the cytotoxic function (Figure 2A–C) and slightly increased the secretion of IFN-γ and IL-6, especially at high doses (Figure 2D–I). Increased levels of IFN-γ and slight modulations of other secreted factors were also seen in peripheral blood-derived serum when mice received four sequential infusions of sNK cells every 7 days for up to 36 days (Table 1). Although there was a slight modulation of the function of endogenous immune cells post sNK cell infusion, no statistically significant increase was seen, suggesting a minimal chance of cytokine release syndrome with sNK cell therapy.

### 3.3. Infusion of Supercharged NK Cells Inhibited Melanoma Tumor Growth in Hu-BLT Mice

**Figure 3:**
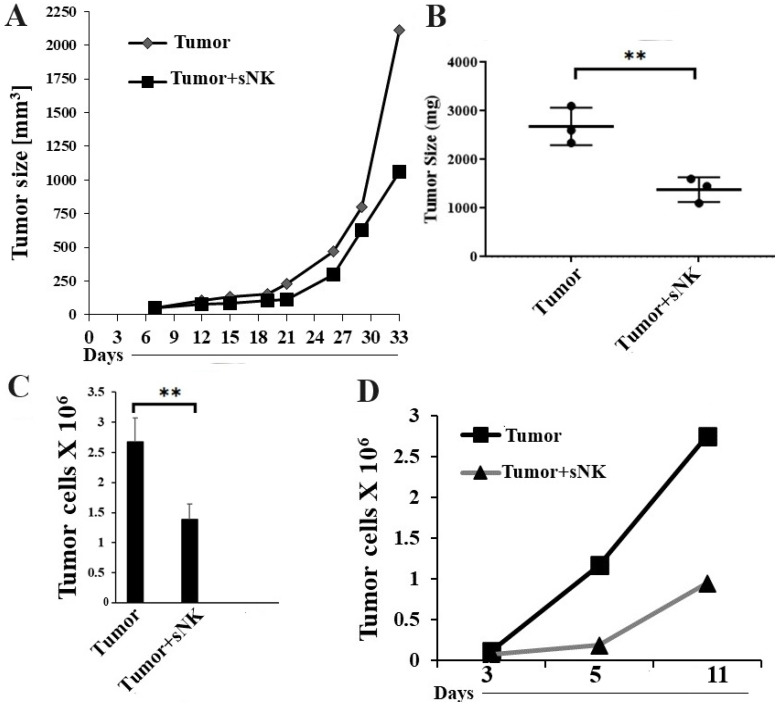
Infusion of sNK cells inhibited tumor growth in melanoma tumor-bearing hu-BLT mice. Hu-BLT mice were injected subcutaneously with 1 × 10^6^ of human A375 melanoma cells in the right flank. One week after tumor implantation, the therapeutic group received a dose of 2 × 10^6^ sNK cells weekly (total of four doses) via tail-vein injection. In vivo tumor size was monitored on the days shown in the figure (**A**). At the end of the experiment, mice were sacrificed, and the tumors were weighed (*n* = 3) (**B**). Tumor samples were dissociated, tumor cells were counted (*n* = 3) (**C**), and ex vivo tumor cultures were performed (**D**). ** (*p*-value 0.001–0.01).

Hu-BLT mice were generated, and human melanoma tumor cells were implanted subcutaneously in the mice as described in the Section 2. In vivo tumor growth was monitored on weekly basis, and we observed a lower tumor growth rate in the mice infused with sNK cells (Figure 3A). Tumor-bearing mice infused with sNK cells did not exhibit morbidity and were able to climb in the cage, whereas untreated tumor-bearing mice became morbid and had complications in climbing and running. The tumor-inhibiting potential of sNK cells was also validated by the small tumor size in the therapeutic group post sacrifice (Figure 3B). When we dissociated the tumor, a higher number of tumor cells (Figure 3C) and increased ex vivo tumor growth (Figure 3D) were observed in the tumors resected from untreated mice compared to the sNK cell-treated group.

### 3.4. sNK Cells Circulated Through PBMCs, the Spleen, and Bone Marrow; Were Found Within the Tumors of Hu-BLT Mice; and Were Responsible for the Induction of In Vivo Differentiation of Melanoma Tumors

**Figure 4:**
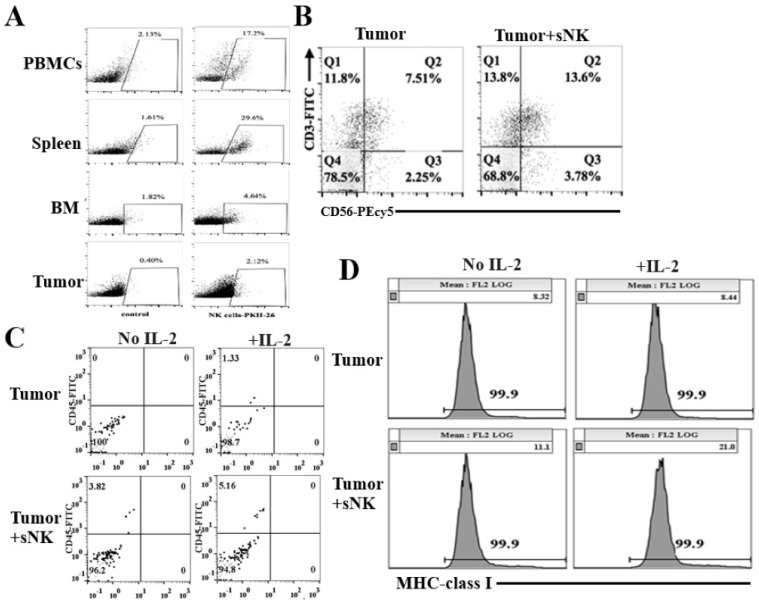
Infused sNK cells distributed to tissues and tumors, resulting in vivo melanoma tumor differentiation in hu-BLT mice. Hu-BLT mice were implanted with melanoma tumors and injected with NK cells as described in Figure 3. sNK celled were PKH-labeled before infusion. At the end of the experiment, mice were sacrificed, and reconstitution of sNK cells was determined in PBMCs spleen, BM, and tumor samples using flow cytometry (**A**). Dissected tumor samples from hu-BLT mice were dissociated, and surface expression of human CD3 and CD56 was determined using antibody staining followed by flow cytometric analysis (**B**). Single cell suspensions (dissociated tumors) of the tumors from each group were cultured untreated or treated with IL-2 (1000 U/mL) (3 × 10^6^ cells/mL) for 7 days. The percentages of infiltrating hu-CD45^+^ immune cells within the non-attached cells (**C**) and MHC class I on attached cells (**D**) on day 7 of culture were determined using antibody staining followed by flow cytometric analysis.

In order to track sNK cells in mouse tissues, sNK cells were labeled with PKH dye before infusion. Post sacrifice, we detected sNK cells in peripheral blood (17.2%), spleen (29.6%), bone marrow (4.64%), and tumor (2.12%) samples (Figure 4A). When tumor-infiltrating human NK and T cells were assessed, increased percentages of NK and NKT cells were found to have infiltrated the tumors of tumor-bearing mice infused with sNK cells when compared to untreated tumor-bearing mice (Figure 4B). Tumors were cultured ex vivo for 7 days, and increased percentages of human CD45+ immune cells were seen in both untreated and IL-2-treated tumors from tumor-bearing mice with sNK infusion compared to untreated tumor-bearing mice (Figure 4C). We also examined the surface expression of MHC class I differentiation antigens on tumors and found higher MHC-class I surface expression on tumors obtained from sNK cell-infused tumor-bearing mice as compared to untreated tumor-bearing mice (Figure 4D).

### 3.5. sNK Cell Infusion Increased Percentages of NK Cells and Restored IFN-γ Secretion Within the Tissue Compartments of Melanoma Tumor-Bearing Mice

**Figure 5:**
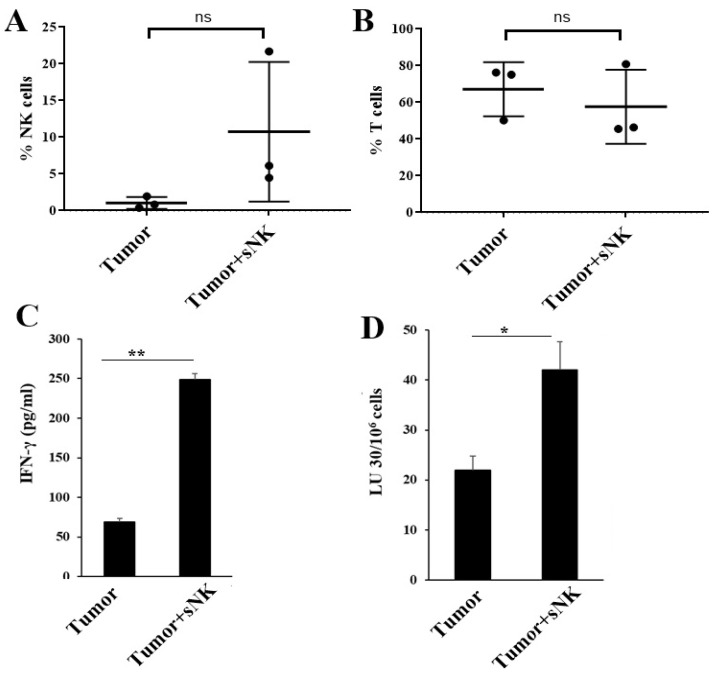
Infusion of sNK cells increased the percentages of endogenous NK cells and restored IFN-γ secretion and NK cell-mediated cytotoxic function in the peripheral blood of melanoma tumor-bearing hu-BLT mice. Hu-BLT mice were implanted with melanoma tumors and injected with NK cells as described in Figure 3. Following sacrifice, peripheral blood was collected, single cell suspensions were prepared, and surface expression of human CD16+CD56+ (*n* = 3) (**A**) and CD3 (*n* = 3) (**B**) was determined using antibody staining followed by flow cytometric analysis. Peripheral blood-derived single cell suspensions were treated with IL-2 (1000 U/mL) and were cultured for 7 days, after which the supernatants were harvested and the levels of IFN-γ were determined using specific ELISAs (**C**). Peripheral blood-derived single cell suspensions were treated with IL-2 (1000 U/mL) and cultured for 7 days, cytotoxicity assays were performed using a standard 4 h ^51^Cr release assay against OSCSCs, and the LU 30/10^6^ cells were determined using an inverse number of cells required to lyse 30% of OSCSCs x100 (**D**). One of three representative experiments is shown in (**C**,**D**). ** (*p*-value 0.001–0.01), * (*p*-value 0.01–0.05) and ^ns^ (*p*-value > 0.05).

**Figure 6:**
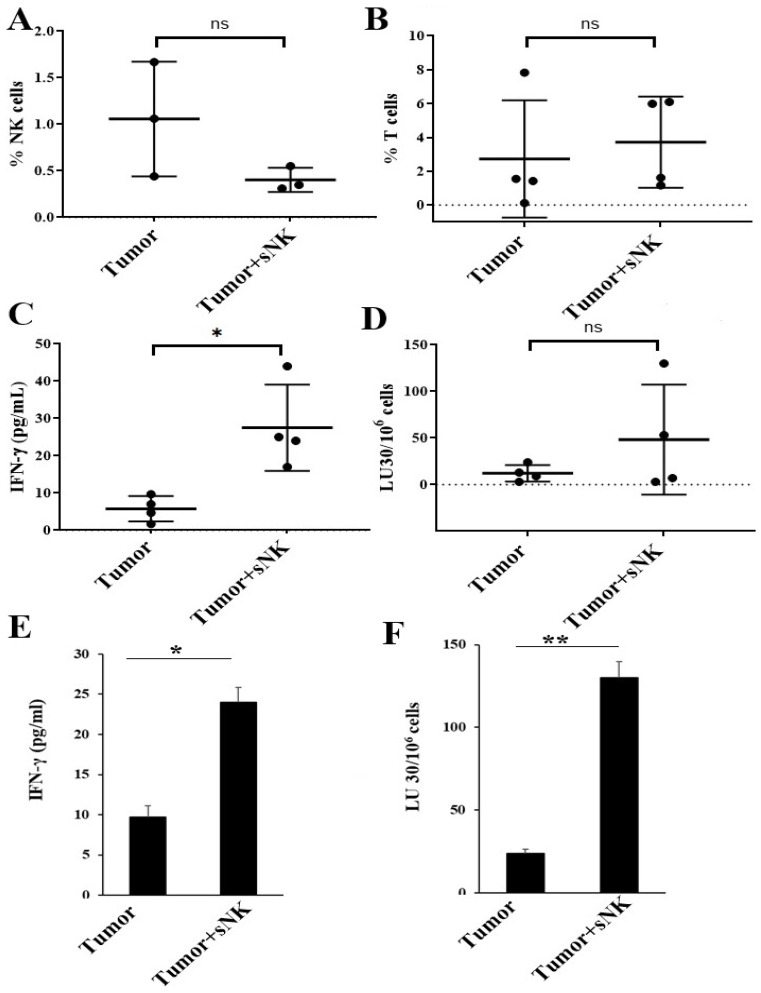
Infusion of sNK cells restored IFN-γ secretion and NK cell-mediated cytotoxic function in the bone marrow of melanoma tumor-bearing hu-BLT mice. Hu-BLT mice were implanted with melanoma tumors and injected with NK cells as described in Figure 3. Following sacrifice, bone marrow was collected, single cell suspensions were prepared, and surface expression of human CD16+CD56+ (*n* = 3) (**A**) and CD3 (*n* = 3) (**B**) was determined using antibody staining followed by flow cytometric analysis. Bone marrow-derived single cell suspensions were treated with IL-2 (1000 U/mL) and cultured for 7 days, after which the supernatants were harvested and the levels of IFN-γ were determined using specific ELISAs. The average of two to three replicates was used in each mouse (each dot), and four animals are shown in the figure (*n* = 4) (**C**). Bone marrow-derived single cell suspensions were treated with IL-2 (1000 U/mL) and cultured for 7 days, cytotoxicity assays were performed using a standard 4 h ^51^Cr release assay against OSCSCs, and the LU 30/10^6^ cells were determined using an inverse number of cells required to lyse 30% of OSCSCs x100. The average of two to three replicates was used in each mouse (each dot), and four animals are shown in the figure (*n* = 4) (**D**). One of three representative experiments is shown in (**E**,**F**). ** (*p*-value 0.001–0.01), * (*p*-value 0.01–0.05), and ^ns^ (*p*-value > 0.05).

**Figure 7:**
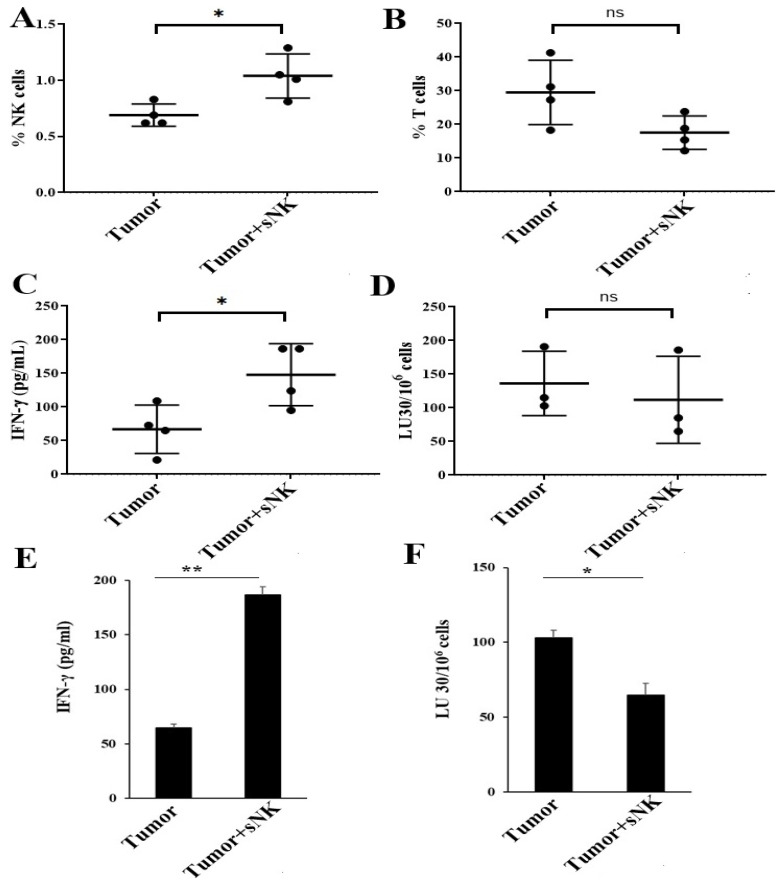
Infusion of sNK cells increased the percentages of endogenous NK cells and restored IFN-γ secretion and NK cell-mediated cytotoxic function in the spleen of melanoma tumor-bearing hu-BLT mice. Hu-BLT were implanted with melanoma tumors and injected with NK cells as described in Figure 3. Following sacrifice, spleen samples were collected, single cell suspensions were prepared, and the surface expression of human CD16+CD56+ (*n* = 4) (**A**) and CD3 (*n* = 4) (**B**) was determined using antibody staining followed by flow cytometric analysis. Spleen-derived single cell suspensions were treated with IL-2 (1000 U/mL) and were cultured for 7 days, after which the supernatants were harvested and the levels of IFN-γ were determined using specific ELISAs. The average of two to three replicates was used in each mouse (each dot), and four animals are shown in the figure (*n* = 4) (**C**). Spleen-derived single cell suspensions were treated with IL-2 (1000 U/mL) and cultured for 7 days, cytotoxicity assays were performed using a standard 4 h ^51^Cr release assay against OSCSCs, and the LU 30/10^6^ cells were determined using an inverse number of cells required to lyse 30% of OSCSCs x100. The average of two to three replicates was used in each mouse (each dot), and three animals are shown in the figure (*n* = 3) (**D**). One of three representative experiments is shown in (**E**,**F**). ** (*p*-value 0.001–0.01), * (*p*-value 0.01–0.05), and ^ns^ (*p*-value > 0.05).

Higher NK cell percentages in PBMCs (Figure 5A) were seen in tumor-bearing hu-BLT mice infused with sNK cells. There was no change or a slight decrease in the percentages of T cells in the peripheral blood (Figure 5B). A different profile of NK and T-cell percentages was seen in the BM of tumor-bearing mice injected with sNK cells. There was a slight decrease in NK cell percentages, whereas a slight increase in T-cell percentages was observed (Figure 6A,B) in tumor-bearing mice injected with sNK cells. Increased secretion of IFN-γ was observed in peripheral blood-derived serum (Table 2), PBMCs (Figure 5C and Figure S2A, and Table 3), and BM (Figure 6C,E) of tumor-bearing mice injected with sNK cells. Higher NK cell percentages, decreased T cell percentages, and increased IFN-γ secretion were seen in spleen of tumor-bearing hu-BLT mice infused with sNK cells (Figure 7A–C,E and Table 4). Increased secretion of other factors, with the exception of IL-10, was seen in peripheral blood-derived serum (Table 2). PBMCs expressed increased secretion of factors except VEGF (Table 3). Spleen samples from tumor-bearing mice injected with sNK demonstrated increased secretion of factors determined in this study (Table 4).

Increased NK cell-mediated cytotoxicity was observed in PBMCs (Figure 5D and Figure S2B) and BM-derived immune cells (Figure 6D,F) from tumor-bearing mice infused with sNK cells. A different NK cell-mediated cytotoxicity profile was seen in the spleen; sNK cell-infused groups showed decreased levels of NK cell-mediated cytotoxicity compared to the untreated tumor-bearing group (Figure 7D,F).

### 3.6. sNK Cell Infusion Restored IFN-γ Secretion in NK and T Cells of Melanoma Tumor-Bearing Mice

**Figure 8:**
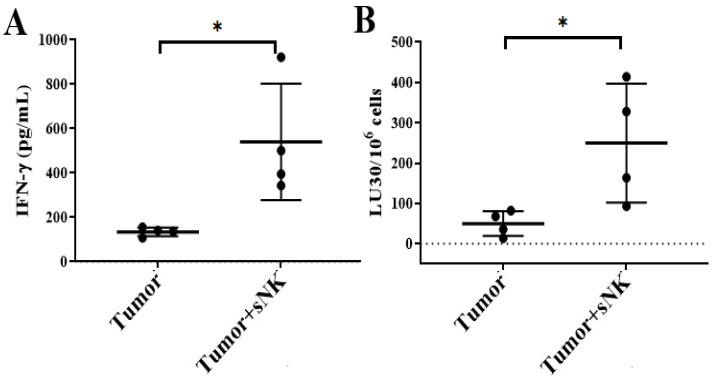
Infusion of sNK cells restored IFN-γ secretion and NK cell-mediated cytotoxic function in the spleen-derived NK cells of melanoma tumor-bearing hu-BLT mice. Hu-BLT were implanted with melanoma tumors and injected with NK cells as described in Figure 3. Following sacrifice, the spleen was collected, single cell suspensions were prepared, and NK cells were purified. Spleen-derived NK cells were treated with IL-2 (1000 U/mL) and were cultured for 7 days, after which the supernatants were harvested and the levels of IFN-γ were determined using specific ELISAs. The average of two to three replicates was used in each mouse (each dot), and four animals are shown in the figure (*n* = 4) (**A**). Spleen-derived NK cells were treated with IL-2 (1000 U/mL) and cultured for 7 days, cytotoxicity assays were performed using a standard 4 h ^51^Cr release assay against OSCSCs, and the LU 30/10^6^ cells were determined using an inverse number of cells required to lyse 30% of OSCSCs x100. The average of two to three replicates was used in each mouse (each dot), and four animals are shown in the figure (*n* = 4) (**B**). * (*p*-value 0.01–0.05).

The sNK cell-infused group also expressed an increased level of IFN-γ in spleen-derived NK (Figure 8A) and T (Figure S3) cells, in addition to expressing increased NK cell-mediated activity in spleen-derived NK cells (Figure 8B). Overall, restored immune function was observed following sNK cell therapy in melanoma tumor-bearing mice.

## 4. Discussion

Melanoma tumors can escape NK cells’ recognition by cleaving and shedding the soluble forms of NKG2L, such as MICA/B and ULBPs [74,75]. The binding of soluble NKG2DL to NKG2D on NK cells reduces NK cells’ ability to recognize and kill tumor cells [76,77]. Melanoma and the melanoma tumor microenvironment secret immunosuppressive molecules and factors such as IDO, PGE2, TGF-β, prostaglandins, and IL-10, which can downmodulate NK cell-activating receptors and can inhibit NK cell activity [34,78]. However, supercharged NK (sNK) cells express a memory-like phenotype and activating receptors, including NKG2D, and they may have the potential to overcome these tumor escape strategies of melanoma [34]. When supernatants of primary NK cells and sNK cells were used to treat melanoma tumors, sNK supernatants induced a higher increase of MICA/B on tumors compared to the primary NK cell supernatants (Figure S1). Thus, an increase in the number of NKG2D receptors on sNK cells and increased MICA/B on melanoma tumors during sNK cell interaction could contribute to increased efficacy of sNK cells against melanoma tumors compared to primary NK cells. Our studies uncovered intriguing findings about the exceptional abilities of sNK cells. These cells exhibit remarkable resilience by preserving vital activating surface receptors, cytokine secretions, and cytotoxic functions [63]. Their unique capacity to target both stem-like and differentiated tumors [68] highlights their superior effectiveness in combating a wide range of tumors over prolonged periods, demonstrating their potent anti-tumor capabilities.

We have previously shown that a single infusion of sNK cells is able to significantly decrease or eliminate oral, pancreatic, and uterine cancers in hu-BLT mice [79,80]. In this report, we extended those studies to melanoma tumors to see if there were any differences in response to sNK cells. In addition, we studied not only the effect of sNK cell therapy within the tumor microenvironment but also its effect on PBMCs, bone marrow, and the spleen. Infusion of sNK cells resulted in increased numbers of circulating PKH-stained sNK cells in PBMCs, the spleen, bone marrow, and the tumor (Figure 3A). Interestingly, the levels of sNK cells were the highest in the spleen, followed by PBMCs, and lowest in bone marrow. There were increased levels of sNK cells in tumor cells, although the percentages were lower in comparison to the other tissues (Figure 3A). The biodistribution of sNK cells was similar between the disease-free (Figure 1) and tumor-bearing mice (Figure 3A). These results indicate that the presence of a tumor did not cause significant changes in sNK cell distribution in the tissues. In both cases, a minimal biodistribution of sNK cells was seen in the bone marrow; this may be due to sNK cells’ interaction with the stromal bone marrow niche. It was found that stromal cells negatively impact NK cell activation, cytokine release, and cytotoxic activity against cancer [81,82].

Infusion of sNK cells significantly decreased the size and the weight of the tumor and delayed the growth of tumors (Figure 3). When single cells were prepared from the tumors and equal numbers of tumors from both groups were cultured for several days, much less tumor growth was seen in the tumor cells from tumor-implanted mice treated with sNK cells as compared to the untreated group. We then focused on the type of immune cells circulating in the tumor cells. After single-cell preparation of the tumors, the levels of CD3 and CD56 subpopulations were determined in the tumor microenvironment. There was an increased percentage of CD3-CD56+ NK cells, in addition to an increase in total CD3+ T cells and CD3+CD56+ NKT cells within the tumor cells (Figure 4B). We then cultured the tumor cells with and without IL-2 treatment and measured the circulating CD45+ immune cells. The proportion of CD45+ immune cells was higher in sNK-infused tumor-implanted BLT mice as compared to mice implanted with tumors alone, and the addition of IL-2 further increased the levels of CD45+ immune cells within the tumor cultures. We have previously shown sNK-mediated increase in NK cells and subsequent induction of IFN-γ increased the levels of MHC class I receptors in tumor cells. Accordingly, we observed higher expression of MHC class I receptors in tumors resected from hu-BLT mice infused with sNK cells. Treatment of tumors resected from tumor-implanted hu-BLT mice infused with sNK and treated with IL-2 substantially increased the levels of MHC class I expression when compared to mice implanted with tumors alone (Figure 4D). These studies indicate that sNK cells circulate within the tumor and are likely responsible in part for the increased MHC class I levels in tumors, which we have shown to be part of the four major biomarkers indicating the levels of differentiation of tumor cells [79,80].

We then assessed the levels of circulating NK cells in PBMCs and found that the levels of NK cells were elevated in tumor-implanted mice infused with sNK cells. Due to the higher variability between mice, we could not show statistical significance, even though, on average, the levels were much higher in tumor-implanted mice infused with sNK cells as compared to mice implanted with tumors only. This observation is very important, since we see the same trend in human patients infused with sNK cells, in which case the percentages of NK cells rise significantly and remain high for greater than 1 year (manuscript submitted). Based on chip analysis in humans, the elevated NK cells are from the recipient (autologous) rather than the donor’s sNK cells. In this study, the donor’s sNK cells were from male donors, and the recipient was female; therefore, in the chip assay, we could only see the X chromosome and not the Y chromosome, indicating that the elevated percentages of NK cells were autologous and not allogeneic. We then assessed the levels of IFN-γ and NK cell-mediated cytotoxicity secreted by PBMCs and found significantly higher levels of IFN-γ and cytotoxicity against NK-specific targets (Figure 5C,D and Figure S2). Interestingly, no difference could be seen for the levels of T cells within PBMCs of mice implanted with a tumor and infused with sNK cells as compared to mice implanted with tumors alone. In addition, when other cytokines and chemokines were assessed in supernatants recovered from PBMCs, we not only observed increased IFN-γ and TNF-α release but also the upregulation of many other cytokines and chemokines in the tumors of mice infused with sNK cells as compared mice implanted with tumors alone (Table 3). We have previously shown that the combination of IFN-γ and TNF-α elevates MHC class I, CD54, and PDL1, whereas it decreases CD44 expression, a profile which was used to distinguish between stem-like cells and their differentiated counterparts [83]. Differentiation of tumor cells with IFN-γ and TNF-α was found to slow tumor growth substantially [83].

In addition to PBMCs, we also observed similar patterns of increased cytokine and chemokine release in the serum of tumor-implanted hu-BLT mice that received an infusion of sNK cells as compared to mice implanted with tumors alone (Table 2). Both IFN-γ and TNF-α release were upregulated in tumor mice receiving sNK cells (Table 2). In contrast, the levels of circulating NK cells in bone marrow were, on average, lower in tumor mice infused with sNK cells (Figure 6A). At the moment, it is unclear why bone marrow acts differently from PBMC and spleen samples (Figure 5, Figure 6 and Figure 7). However, in accordance with PBMC and spleen samples, the bone marrow cells exhibited significantly increased secretion of IFN-γ and higher NK cell-mediated cytotoxicity in the tumor-bearing mice infused with sNK cells as compared to mice implanted with tumors alone (Figure 5, Figure 6 and Figure 7). The spleen exhibited higher percentages of NK cells in the tumor-bearing mice infused with sNK cells, and the cells mediated increased secretion of IFN-γ while mediating reduced cytotoxicity as compared to mice implanted with tumors alone (Figure 7). Similarly, we saw higher secretion of cytokines and chemokines from splenocytes when assessed by multiplex assay (Table 4).

We then sorted the NK cells and T cells from the spleens of the mice and performed functional assays. We observed significantly increased secretion of IFN-γ and augmented cytotoxicity in spleen-derived NK cells of tumor-bearing mice infused with the sNK cells (Figure 8). Likewise, sorted T cells were also able to increase the levels of IFN-γ from the tumors of mice infused with sNK cells, although due to high variability between mice, we could not achieve statistical significance (Figure S3). We have previously shown that sNK cells preferentially expand CD8+ T cells and increase their functional capabilities [49,80]. Thus, sNK cells increase the number cytotoxic T cells and also increase the regulatory function of T cells in vivo.

## 5. Conclusions

This study showed the efficacy of sNK cells in inhibiting melanoma growth and to restoring inhibited immune cell function in tumor-bearing mice. We previously investigated the combination of sNK cell therapy with either chemotherapy or checkpoint inhibitors in oral and pancreatic tumor-bearing hu-BLT mice and observed slightly better efficacy compared to sNK cells alone with chemotherapy and checkpoint inhibitors. These combinations are under investigation for melanoma-bearing hu-BLT mice. Previous research revealed the potent anti-cancer impact of sNK cells in glioblastoma 2D and 3D tumor spheroids [84]. This showcases sNK cell-driven cytotoxicity within a realistic immunosuppressive setting. The next crucial step is to replicate these studies in a melanoma tumor model. The use of sNK cells in clinical trials has yet to receive FDA approval in the United States, but they are in use in palliative care in Mexico. The production, analysis, and verification of the product’s quality were performed per standardized procedures consistent with current good manufacturing practices (cGMP) for biological products. Product feasibility, scale-up, and batch–batch standardization were validated as per regulatory considerations [85].

## Figures and Tables

**Table 1:** sNK cells increased cytokine and chemokine secretions from the peripheral blood-derived serum of the disease-free hu-BLT mice. Hu-BLT mice were adoptively transferred with four sNK cell doses every 7 days: 1 × 10^6^ and 2 × 10^6^ sNK cells for 36 days. Following the sacrifice, peripheral blood was collected to harvest sera. Sera were analyzed for identification of secreted factors using multiplex arrays.

	IFN-γ	ITAC	GMCSF	FRACTALKINE	IL-10	MIP-3a	IL-12	IL-13	IL-17A	IL-1b	IL-21	IL-4	IL-23	MIP-1a	IL-8
Hu-BLT	7	34	0	0	0	0	0	0	2	0	0	0	0	0	3
+sNK (1 million)	12	17	8	28	1	17	2	4	18	3	11	4	26	5	36
+sNK (2 million)	18	13	11	37	3	23	7	3	21	7	9	9	88	7	58

**Table 2:** Infusion of sNK cells restored secretion of IFN-γ and other factors in the peripheral blood-derived serum of melanoma tumor-bearing hu-BLT mice. Hu-BLT mice were injected subcutaneously with 1 × 10^6^ of human A375 melanoma cells in the right flank. One week after tumor implantation, the therapeutic group received 2 × 10^6^ sNK cells via tail-vein injection. Animals were sacrificed at the endpoint; serum from peripheral blood was obtained as described in the Section 2; and multiplex arrays were performed to determine secretion of cytokines, chemokines, and growth factors. The average of two to three replicates was used in each mouse (each dot), and four animals are shown in the figure (*n* = 1–3).

Serum	IFN-γ	TNF-α	IL-10	IL-12	IL-17A	IL-1b	IL-2
Tumor	5.5 ± 3	7 ± 1.4	33.5 ± 16	3 ± 1.4	6 ± 0.1	2 ± 1.1	5 ± 2.1
Tumor + sNK	60	26	17	24	48	16	16
**Serum**	**GMCSF**	**MIP-1b**	**ITAC**	**Fractalkine**	**MIP-3A**		
Tumor	291.5 ± 168	6.5 ± 0.7	16 ± 7	109 ± 12	4 ± 1.1		
Tumor + sNK	328	33	32	320	27		

**Table 3:** Infusion of sNK cells restored secretion of IFN-γ and other factors in the peripheral blood of melanoma tumor-bearing hu-BLT mice. Hu-BLT mice were implanted with human A375 melanoma and sNK cells as described in Table 2. Following sacrifice, peripheral blood was collected, and single cell suspensions were prepared and treated with IL-2 (1000 U/mL) for 7 days, after which the supernatants were harvested and multiplex arrays were performed to determine the secretion of cytokines, chemokines, and growth factors. The average of two to three replicates was used in each mouse (each dot), and four animals are shown in the figure (*n* = 4).

PBMCs	IFN-γ	TNF-α	IL-6	IFN-a	VEGF	GMCSF	IP-10	Eotaxin	Fractalkine
Tumor	0.9 ± 0.7	2.5 ± 1.6	5 ± 3.3	10 ± 5.8	57 ± 10	0.5 ± 0.5	3.2 ± 0.1	3.8 ± 1	14 ± 5
Tumor + sNK	2 ± 0.7	4.1 ± 3	7.7 ± 2.3	24 ± 2	33.6 ± 12	3.1 ± 1.9	6 ± 2.5	4.3 ± 0.6	22 ± 7

**Table 4:** Infusion of sNK cells restored the secretion of IFN-γ and other factors in the spleen of melanoma tumor-bearing hu-BLT mice. Hu-BLT mice were implanted with human A375 melanoma and sNK cells as described in Table 2. Following sacrifice, spleen samples were collected, and single cell suspensions were prepared and treated with IL-2 (1000 U/mL) for 7 days, after which the supernatants were harvested and multiplex arrays were performed to determine the secretion of cytokines, chemokines, and growth factors. The average of two replicates was used in each mouse (each dot), and four animals are shown in the figure (*n* = 4).

Spleen	IFN-γ	TNF-α	IL-6	IL-10	GMCSF	MCP-1	MIP-1a	MIP-1b	MDC	IP-10
Tumor	3 ± 2	3.7 ± 1.2	1.9 ± 1	2.5 ± 0.6	6.15 ± 2.8	5.3 ± 3.5	202 ± 165	63 ± 17	211 ± 91	14.7 ± 4.5
Tumor + sNK	6.5 ± 2.5	6.9 ± 1.8	4.9 ± 1.1	24 ± 18	12.2 ± 0.1	14.1 ± 2.3	805 ± 296	118 ± 48	405 ± 147	34 ± 21

## Data Availability

The data presented in the study is included in the main and Supplementary Files of this manuscript.

## Supplementary Materials

The following supporting information can be downloaded at: https://www.mdpi.com/article/10.3390/cancers17152430/s1, Figure S1: Increase in MICA/B surface expression in A375 melanoma tumor cells induced by supernatants from supercharged NK cells compared to primary NK cells; Figure S2: Infusion of sNK cells restored IFN-γ secretion and NK cell-mediated cytotoxic function in the peripheral blood of melanoma tumor-bearing hu-BLT mice; Figure S3: Infusion of sNK cells restored IFN-γ secretion in the spleen-derived T cells of melanoma tumor-bearing hu-BLT mice.

## Abbreviations

NK cells	Natural killer cells
sNK cells	Supercharged NK cells
CSCs	Cancer stem-like cells
Hu-BLT	Humanized bone marrow/liver/thymus
IFN-γ	Interferon-gamma
IL-2	Interleukin 2
OCs	Osteoclasts
PBMCs	Peripheral blood-derived mononuclear cells
OSCSCs	Oral squamous cancer stem-like cells
ELISAs	Enzyme-Linked Immunosorbent Assays
Cr	Chromium
