# Supplementary material for: Supercharged Natural Killer (sNK) Cells Inhibit Melanoma Tumor Progression and Restore Endogenous NK Cell Function in Humanized BLT Mice
Source: Cancers (Basel). 2025 Jul 23;17(15):2430. doi: 10.3390/cancers17152430 (PMC12346276; doi:10.3390/cancers17152430)

Supplementary Data

Figure S1: Higher increase of MICA/B surface expression in A375 melanoma tumor cells induced by supernatants from supercharged NK cells compared to primary NK cells
Differentiation of A375 melanoma tumors was conducted with an average total of 15000 pg IFN-γ. sNK cells were generated as described in Materials and Methods. on day 15 of sNK cell cultures, another set of NK cells was isolated from the same healthy individuals and were treated with a combination of IL-2 (1000 U/ml) and anti-CD16 mAb (3 μg/ml) for 18 hours. The supernatants were harvested from primary IL-2 and anti-CD16 mAbs-treated NK cells (pNK) and sNK cells to determine IFN-γ secretion using a single ELISA. The volume of NK cell supernatants to treat the tumor cells was determined based on the levels of IFN-γ in the supernatants as detected by ELISA and was divided over 4 days with daily treatment. On day 5, the surface expression levels of MICA/B were assessed using flow cytometric analysis. Isotype control IgG2 was used isotype control.

Figure S2: **Infusion of sNK cells restored IFN-γ secretion and NK cell-mediated cytotoxic function in the peripheral blood of melanoma tumor-bearing hu-BLT mice**

Hu-BLT were implanted with melanoma tumors and injected with NK cells as described in the main file, Figure 1. Following sacrifice, peripheral blood was collected, single cell suspensions were prepared, and were treated with IL-2 (1000 U/ml) and cultured for 7 days, after which the supernatants were harvested and the levels of IFN-γ were determined using specific ELISAs (n=3) **(A)**. Peripheral blood-derived single cell suspensions were treated with IL-2 (1000 U/ml) and were cultured for 7 days, and cytotoxicity assays were performed using a standard 4-hour ^{51}Cr release assay against OSCSCs, and the LU 30/10^6 cells were determined using an inverse number of cells required to lyse 30% of OSCSCs x100 (n=2) **(B)**.

Figure S3. Infusion of sNK cells restored IFN-γ secretion in the spleen-derived T cells of melanoma tumor-bearing hu-BLT mice

Hu-BLT were implanted with melanoma tumors and injected with NK cells as described in main file, Figure 1. Following sacrifice, the spleen was collected, single cell suspensions were prepared, and T cells were purified. Spleen-derived T cells were treated with IL-2 (100 U/ml) and were cultured for 7 days, after which the supernatants were harvested and the levels of IFN-γ were determined using specific ELISAs (n=3).

Figure S1

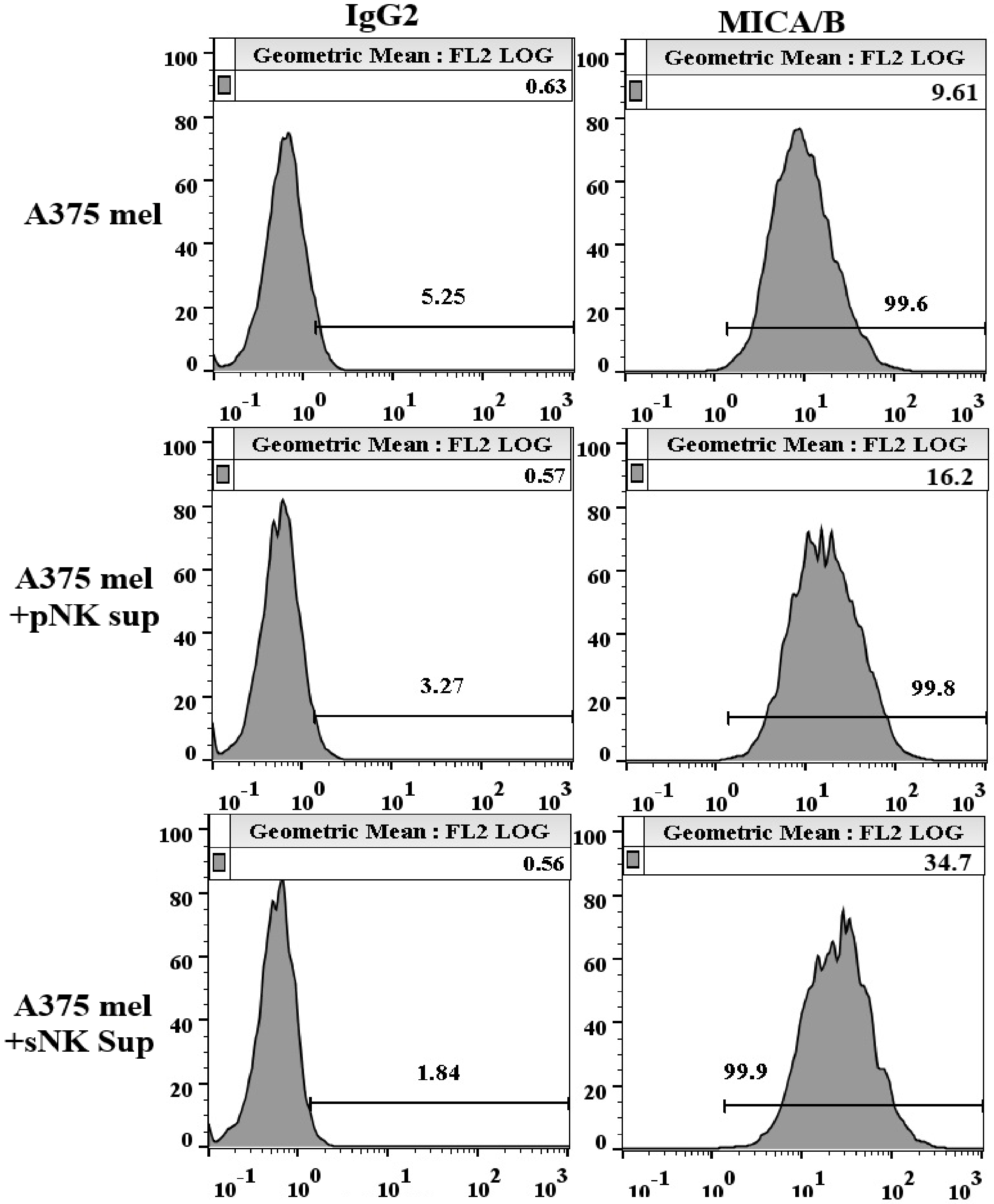
IgG2
MICA/B
A375 mel
A375 mel +pNK sup
A375 mel +sNK Sup
Geometric Mean : FL2 LOG
0.63
9.61
0.57
16.2
0.56
34.7
5.25
99.6
3.27
99.8
1.84
99.9
100
80
60
40
20
0
10^{-1}
10^{0}
10^{1}
10^{2}
10^{3}

Figure S2 PBMCs

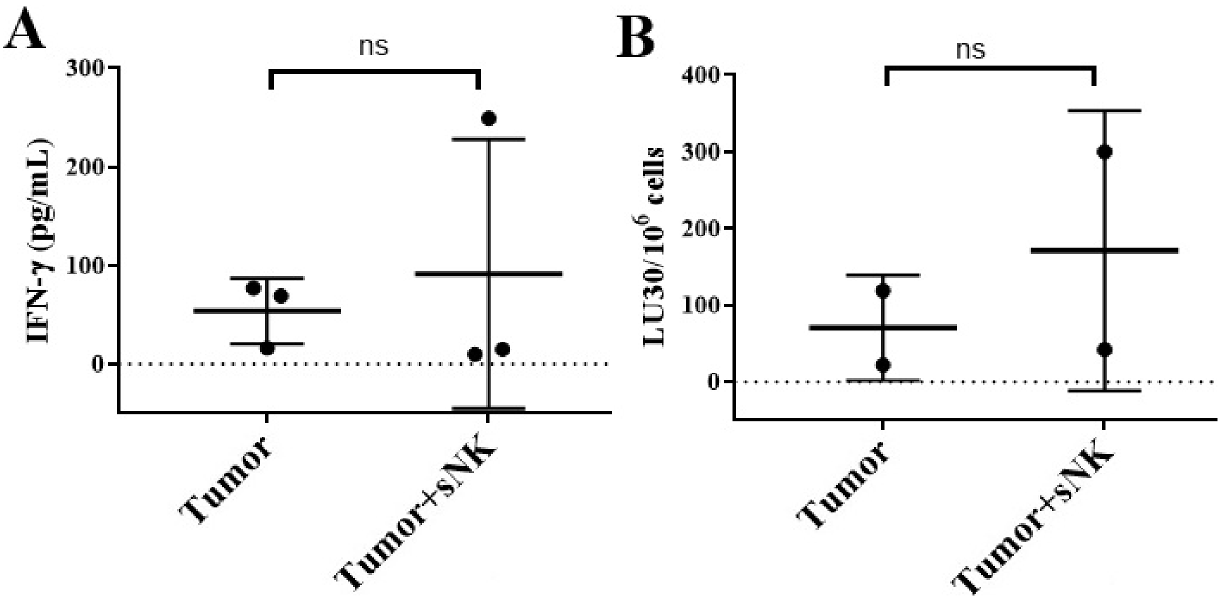

Figure S3 T cells

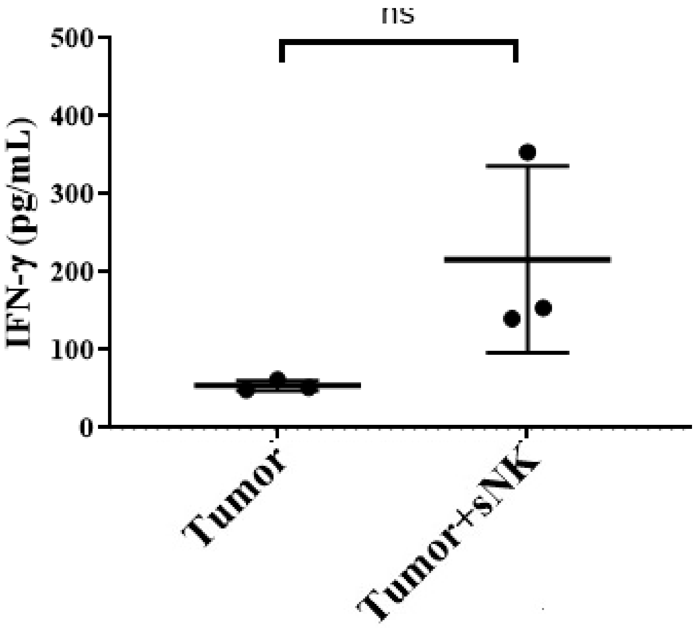

Supplement: Supplementary file 1 [file cancers-17-02430-s001.zip › cancers-3727361-supplementary.pdf]
